# Dynamics of adsorbed polymers on attractive homogeneous surfaces

**DOI:** 10.1038/srep37156

**Published:** 2016-11-16

**Authors:** Qing-Hui Yang, Meng-Bo Luo

**Affiliations:** 1 Department of Physics, Zhejiang University, Hangzhou 310027, China; 2 Collaborative Innovation Center of Advanced Microstructures, Nanjing, China

## Abstract

Dynamic behaviors of polymer chains adsorbed on an attractive, homogeneous surface are studied by using dynamic Monte Carlo simulations. The translational diffusion coefficient *D*_xy_ parallel to the surface decreases as the intra-polymer attraction strength *E*_PP_ or the polymer-surface attraction strength *E*_PS_ increases. The rotational relaxation time *τ*_R_ increases with *E*_PS_, but the dependence of *τ*_R_ on *E*_PP_ is dependent on the adsorption state of the polymer. We find that *τ*_R_ decreases with increasing *E*_PP_ for a partially adsorbed polymer but it increases with *E*_PP_ for a fully adsorbed polymer. Scaling relations *D*_xy_ ~ *N*^−α^ and *τ*_R_ ~ *N*^β^ are found for long polymers. The scaling exponent α is independent of *E*_PS_ for long polymers but increases with *E*_PP_ from α = 1.06 at *E*_PP_ = 0. While β ≈ 2.7 is also roughly independent of *E*_PS_ for the adsorbed polymer at *E*_PP_ = 0, but β increases with *E*_PS_ at *E*_PP_ > 0. Moreover, we find that β always decreases with increasing *E*_PP_. Our results reveal different effects of the attractive surface on the diffusion and rotation of adsorbed polymers.

Polymers can be adsorbed on surfaces/interfaces through physical adsorption or chemical bonding. Adsorption of flexible polymers can significantly and permanently modify the properties of surfaces^1^. Simultaneously the attractive surface also changes the conformational and dynamic properties of polymers^2^,^3^,^4^. The properties of adsorbed polymers play important roles in many chemical and biological applications^1^,^5^,^6^,^7^, such as polymer nanocomposite materials^8^, compatibilization by copolymers^9^,^10^,^11^, colloid stabilization by polymeric surfactants^12^,^13^, coating and lubrication^14^, and DNA segregating in bacteria^15^ and packaging in viruses^16^. The adsorption of polymers on surfaces/interfaces and the dynamical properties of adsorbed polymers have motivated extensive studies in experiment^3^,^17^,^18^,^19^,^20^,^21^,^22^,^23^,^24^,^25^, theory and simulation^2^,^4^,^26^,^27^,^28^,^29^,^30^,^31^,^32^,^33^,^34^,^35^,^36^,^37^,^38^,^39^,^40^,^41^,^42^,^43^,^44^,^45^,^46^,^47^,^48^,^49^.

The self-avoiding walk (SAW) model polymer on the simple cubic (sc) lattice was extensively used for studying the adsorption of polymers in theory as well as in simulations^27^. The surface is usually simply assumed to be infinitely large, flat, and homogeneous. In this model system, every walk contacting with the surface is assigned a polymer-surface interacting energy –*E*_PS_. With increasing the surface attraction strength *E*_PS_ (the same as decreasing temperature), the polymer exhibits a phase transition from a desorbed state to an adsorbed state at the critical attraction (temperature). The critical attraction (temperature) was usually named as the critical adsorption point (CAP)^27^,^31^,^35^,^41^, at which the free energy of an adsorbed polymer is equal to that of a free polymer in solution^26^,^37^. At temperature below the CAP, the polymer is partially adsorbed on the surface and the dynamics of the polymer is confined. While at very low temperature far below the CAP, the polymer can be regarded as a fully adsorbed polymer^30^. The fully adsorbed polymer can be simply regarded as a two-dimensional (2D) polymer, while the desorbed polymer at temperature above the CAP can be regarded as a free polymer in three-dimensional (3D) dilute solution.

It was found that the dynamic properties of a polymer, such as self-diffusion coefficient and rotational relaxation time, were significantly influenced by the attractive surfaces. The translational diffusion parallel to the surface was slowed down obviously after the polymer was adsorbed on the surface^25^,^30^,^31^,^32^,^50^. However, there were contradictory results about the scaling exponent α of the translational diffusion coefficient *D*_xy_ with polymer length *N, D*_xy_ ~ *N*^−α^. It was pointed out that the exponent α was dependent on system temperature for the adsorbed SAW lattice polymer in the absence of intra-polymer attraction, specifically α was changed from 1 for partially adsorbed polymers to 2 for fully adsorbed ones^30^. However, simulation on an off-lattice polymer model showed that α was roughly independent of the attraction strength of the surface for the polymer in the absence of intra-polymer attraction^31^. In that case α ≈ 1.1 was estimated for adsorbed polymers^31^. While α ≈ 1.1 was also found for both desorbed polymer and fully adsorbed polymer using lattice polymer model^32^. But a small value of α ≈ 0.8 was estimated for a partially adsorbed SAW polymer at temperature near CAP^32^. Experiment on surface diffusion of Poly(ethylene glycol) found that the diffusion of polymers was slowed down and the value of α was increased obviously after adsorption^22^. Similarly, experiment showed that DNA diffused slower in agarose gels than in solution, and α was increased obviously by confinements^51^.

On the other hand, studies have revealed that the rotational relaxation time *τ*_R_ of a polymer became larger when the polymer was end-grafted^33^,^52^ or adsorbed on surfaces^25^,^30^,^31^,^32^,^43^. It was found that *τ*_R_ increased with the attraction strength of surface for the partially adsorbed polymers^30^,^31^,^32^,^43^. In dilute solution the SAW polymer chain follows Rouse dynamics as *τ*_R_ ~ *N*^β^ with the exponent β = 1 + 2ν = 2.2. Hahn and Kovac found that the presence of an impenetrable surface did not change the scaling of the rotational relaxation times with chain length for end-grafted polymers^52^. Although *τ*_R_ was increased with a decrease in the temperature, β was found to be roughly independent of temperature^30^. While simulation on BF polymer showed that for a partially adsorbed polymer β lied in the range 2.2 < β < 2.5, that is, as the temperature fell through the adsorption transition, a smooth crossover from 3D to 2D dynamics took place^32^. Off-lattice Monte Carlo (MC) simulation found that the relaxation time of the partially adsorbed polymer could be scaled with a large β ≈ 2.65^31^. However, there is no experimental data about the behavior of β for adsorbed polymers.

In addition to the polymer-surface attraction *E*_PS_, the intra-polymer attraction *E*_PP_ is usually taken into account for more realistic polymer model. When two non-bonded monomers locate at nearest neighbor (NN) sites on the lattice, an attractive energy −*E*_PP_ is assigned. With an increase in the intra-polymer attraction strength *E*_PP_, polymer exhibits a phase transition from an extended coil conformation with large statistical size to a compact globule with small statistical size. Therefore rich phases were found for the adsorbed polymer on surfaces after *E*_PP_ was taken into account^41^,^53^,^54^. However, the dynamics of the adsorbed polymer is not fully understood, especially when the intra-polymer interaction is taken into account.

In this work, we present our study on the dynamics of a physically adsorbed polymer on an attractive, homogeneous surface using dynamic MC simulation. We mainly study the influence of *E*_PS_ and *E*_PP_ on the translational diffusion and rotational relaxation of the adsorbed polymer. Results show that both the translational diffusion coefficient *D*_xy_ parallel to the surface and the rotational relaxation time *τ*_R_ are dependent on *E*_PS_ and *E*_PP_. We find that *D*_xy_ decreases with increasing *E*_PS_ or *E*_PP_. The scaling exponent α in *D*_xy_ ~ *N*^−α^ is roughly independent of *E*_PS_ for long polymers, but increases with *E*_PP_. However the behavior of *τ*_R_ is much more complicated. Although *τ*_R_ always increases with *E*_PS_, it decreases with increasing *E*_PP_ for the partially adsorbed polymer and increases with *E*_PP_ for the fully adsorbed polymer. And the scaling exponent β in *τ*_R_ ~ *N*^β^ always decreases with increasing *E*_PP_. We find that β is roughly independent of *E*_PS_ for the adsorbed polymer at *E*_PP_ = 0, but β increases with *E*_PS_ at *E*_PP_ > 0.

## Simulation model and method

### Model

We consider a coarse-grained linear polymer model on the sc lattice. The simulation box is a cuboid with the length *L*_x_, *L*_y_, and *L*_z_ in *x, y*, and *z* directions, respectively. The polymer chain of length *N* is composed of *N* identical monomers numbered sequentially from 1 to *N*. Here, a monomer corresponds to a small group of atoms instead of a specific atom in the polymer, and a bond represents the linkage between two monomers rather than a specific covalent bond between two atoms. Each monomer occupies one lattice site and each lattice site cannot be occupied simultaneously by two or more monomers. The bond length can be varied among 1, 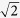, and 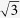 in the present model. In addition to the self-avoiding on the sc lattice, the intra-polymer NN monomer-monomer attraction −*E*_PP_ is considered. In this work *E*_PP_ = 0 means only self-avoiding effect is taken into account for the polymer chain, that corresponding to an ordinary SAW polymer. An additional attraction is introduced in the polymer when *E*_PP_ > 0. And the statistical size of the polymer, e.g. the mean-square radius of gyration 〈*R*_G_^2^〉, is decreased with increasing *E*_PP_.

For the polymer in 3D dilute solution, periodic boundary conditions (PBCs) are adopted in all the three directions. While for the adsorbed polymer, PBCs are only adopted in the *x* and *y* directions. An infinitely large, impenetrable, homogeneous, flat surface is placed at *z* = 0, which produces a homogeneous attraction to the polymer. When monomers are at the NN layer of the surface, i.e. at *z* = 1, an attractive polymer-surface attraction −*E*_PS_ is assigned. Therefore the energy of the polymer can be described as


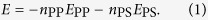


Here *n*_PP_ is the number of intra-polymer NN pairs and *n*_PS_ is the surface contact number of polymer.

### Simulation method

The dynamics of polymer chains is achieved by adopting bond fluctuation method invented by Carmesin and Kremer^55^ and Metropolis algorithm. The global dynamics of polymer chains is achieved by a huge number of local displacements of monomers resulting from random collisions between chain monomers and solvent molecules. For each local displacement, a monomer is chosen randomly and attempted to move one lattice spacing selected randomly from its six NN sites. If the move satisfies: (i) the new site is empty, (ii) bonds don’t intersect, and (iii) bond lengths vary among 1, 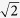, and 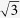, it will be accepted with a probability *p* = min[1, exp(−Δ*E*/*k*_B_*T*)], where Δ*E* is the energy shift due to the change of monomer’s site. In simulation, we count the change of numbers *n*_PP_ and *n*_PS_ for every move, and thus obtain the energy shift Δ*E*. During one MC step (MCS), every monomer attempts to move once on average. In this work, MCS is the unit of time. And the unit of energy is *k*_B_*T* with *k*_B_ the Boltzmann constant and *T* the temperature.

At the beginning of every simulation run, a polymer is generated and equilibrated for about *N*^2.2^ (≈*N*^1+2*ν*^) MCS between a virtual surface at z = *L*_z_/4 and a top surface at z = *L*_z_. The virtual surface is repulsive to polymer. With the virtual surface, polymer is equilibrated before adsorption. Then we remove the virtual surface and let the polymer undergo trial moves until it contacts with the bottom adsorbing surface and settles into the adsorbed equilibrium state. At last we let the polymer random diffuse for sufficiently long time which is used for recording the polymer conformational properties. We perform 5000 independent runs with different initial configurations and random number series. Simulation results are averaged over these 5000 independent samples and the simulation errors in the measured parameters are small.

## Results and Discussions

We study the dynamics of a single adsorbed polymer on a flat surface. Simulations are carried out at *k*_B_*T* = 1 below the CAP of the SAW polymer at *E*_PP_ = 0 and *E*_PS_ = 1, which was estimated to be about 1.625^56^. And it was pointed out that the CAP was increased with an increase in *E*_PP_^48^,^53^. Our simulations are performed at *E*_PP_ ≥ 0 and *E*_PS_ ≥ 1, therefore the polymer is always adsorbed during the whole statistical time^46^. However, at such low temperature, the polymer chain can still diffuse and adjust its configuration^50^. Since the attraction is not strong enough in this work, the number of adsorbed monomers is less than the polymer length, therefore the adsorbed polymer is called as a partially adsorbed polymer.

In addition to the partially adsorbed polymer, there are two other states for polymers, i.e. a fully adsorbed polymer and a desorbed polymer. A fully adsorbed polymer would appear at high *E*_PS_. In the present work a 2D polymer model on surface is adopted as the fully adsorbed polymer. While a desorbed polymer appears at low *E*_PS_, and thus a 3D polymer in dilute solution is adopted for the desorbed polymer.

The polymer also exhibits a coil-globule transition in solution as well as on adsorbing surface^57^,^58^. For the present model, the coil-globule transition is occurred at about *E*_PP_ = 0.5, although the transition point increases slightly with *E*_PS_^48^,^53^,^57^.

### Translational diffusion of partially adsorbed polymer

We have measured the translational diffusion for the adsorbed polymer chain. The diffusion is characterized by the mean-square displacement (MSD) of the center of mass


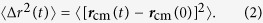


Here < > denotes an ensemble average over independent samples, and ***r***_cm_(*t*) is the position vec*t*or of the center of mass of polymer chain at time *t* and ***r***_cm_(0) at initial time *t* = 0. For the partially adsorbed polymers, the diffusion in the surface normal direction can be neglected and thus the diffusion is mainly confined parallel to the surface. Therefore, we have the parallel component 〈Δ*r*^2^(*t*)〉_xy_ ≈ 〈Δ*r*^2^(*t*)〉 for the partially adsorbed polymers. In this sense, the partially adsorbed polymer can be regarded as a quasi-2D one.

**Figure 1:**
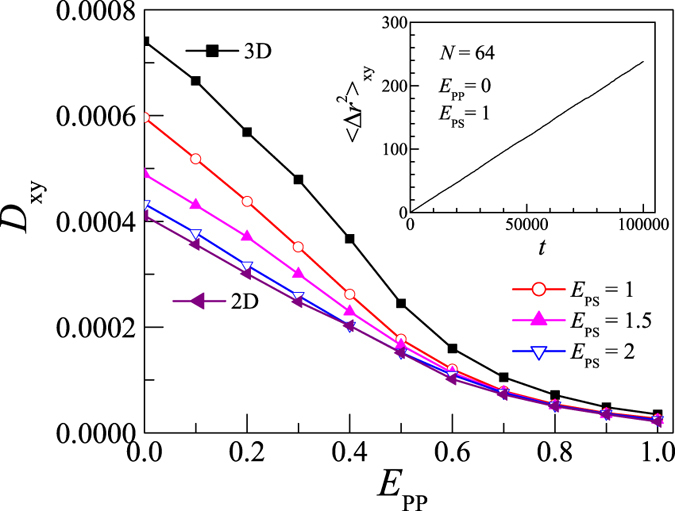
Plot of the translational diffusion coefficient *D*_xy_ versus the intra-polymer attraction *E*_PP_ for polymer in 3D solution, on attractive surfaces at attraction strengths *E*_PS_ = 1, 1.5, and 2, and on 2D surface. Polymer length *N* = 64. The insert shows the dependence of 〈Δ*r*^2^〉_xy_ on time *t* for *N* = 64 at *E*_PP_ = 0 and *E*_PS_ = 1.

Simulation results show that MSD increases linearly with time for the partially adsorbed polymer, analogous to the free polymer in 3D dilute solution. As an example, we show the time dependence of MSD in the inset of Fig. 1. Thus, the translational diffusion coefficient *D*_xy_ for such a quasi-2D system can be obtained through


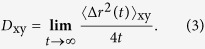


While for the polymer in 3D dilute solution, we have *D*_xy_ = *D* due to the isotropy of our simulation system. We find that the translational diffusion coefficient *D*_xy_ is dependent on the simulation parameters, such as intra-polymer attraction *E*_PP_, polymer-surface attraction *E*_PS_, and polymer length *N*.

We have studied the influence of the intra-polymer attraction *E*_PP_ on the translational diffusion coefficient *D*_xy_. Figure 1 shows the dependence of *D*_xy_ on *E*_PP_ for the polymer adsorbed on surface at *E*_PS_ = 1, 1.5, and 2. We find that the simulated data for *N* = 8, 16, 32, 64, 96, 128 are qualitatively similar, so here we only present the results for *N* = 64. It is clear that *D*_xy_ decreases smoothly with increasing *E*_PP_. It is known that polymer compacts itself or monomers aggregate with the increase in *E*_PP_. This decreases the mobility of monomers and thus decreases the diffusion coefficient of the whole polymer chain.

On the other hand, from Fig. 1 we note that *D*_xy_ also decreases with an increase in the polymer-surface attraction strength *E*_PS_ for all *E*_PP_s. Since the number of adsorbed monomers increases with *E*_PS_, the results indicate that the translational diffusion is obviously slowed down with an increase in the number of adsorbed monomers. Because of confinement introduced by the surface, the mobility of adsorbed monomers is lower than that of desorbed monomers, thus the diffusion coefficient decreases with increasing *E*_PS_.

To further understand the diffusion behavior of the partially adsorbed polymers, the diffusion constants of the polymer in 3D dilute solution and on 2D surface are also calculated. The polymer in the 3D dilute solution corresponds to the utmost limit of weak attraction of the surface and the polymer on the 2D surface corresponds to the utmost limit of strong attraction of the surface. The results of the 3D and 2D diffusions are also presented in Fig. 1. We find that 3D diffusion is faster than 2D one. The reason is that the 2D polymer suffers stronger excluded volume (EV) effect, thus the mobility of 2D polymer is lowered down.

It is clear to see that the diffusion of adsorbed polymer locates between the 3D and 2D cases. That is, the adsorbed polymer diffuses slower than the 3D one in dilute solution, but faster than the 2D one on surface. In other words, the adsorbing surface prevents the diffusion of polymers, and the diffusion becomes slow as the adsorption strength *E*_PS_ increases. However, such a difference in *D*_xy_ dies away with the increase in *E*_PP_. For example, at *E*_PP_ = 1, one can see that *D*_xy_ is roughly independent of *E*_PS_.

**Figure 2:**
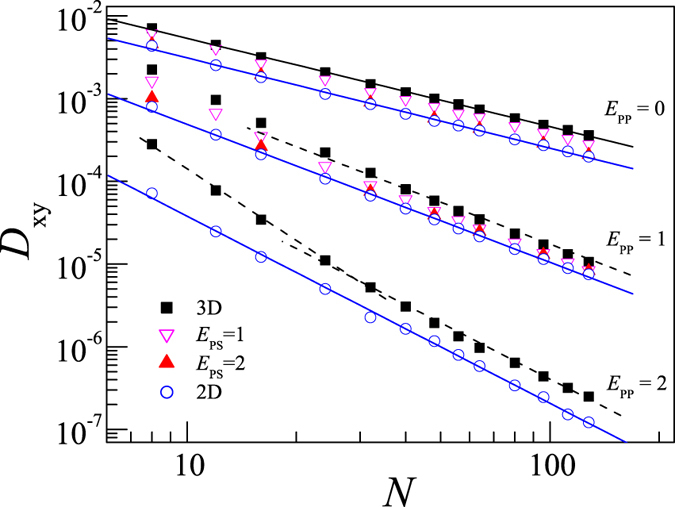
Log-log plot of the translational diffusion coefficient *D*_xy_ versus the polymer chain length *N* for polymers in 3D solution, on attractive surfaces with attractions *E*_PS_ = 1 and 2, and on 2D surface. Three data sets with intra-polymer interactions *E*_PP_ = 0, 1, and 2 are presented. Solid lines are linear fit of simulation data, while dashed lines are guides for eyes.

It was pointed out that the polymer length *N* dependence of *D* scales as *D* ~ *N*^−1^ for the Rouse model when hydrodynamic interactions are ignored for ideal random walk chains^59^. We have checked the scaling relation


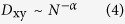


for polymers in different situations. Figure 2 presents the dependence of *D*_xy_ on *N* for polymers in dilute solution, partially adsorbed on surface, and fully adsorbed on surface with different intra-polymer interactions.

For the SAW polymers without intra-polymer attraction (*E*_PP_ = 0), we find that *D*_xy_ can be scaled with *N* in the power law relation *D*_xy_ ~ *N*^−α^ and the apparent exponent is found to be α ≈ 1.06 for all cases, i.e., it is roughly independent of *E*_PS_. The value α ≈ 1.06 indicates that the dynamics of SAW polymers is of Rouse type. It is also in agreement with earlier simulation results for adsorbed polymer chains in good solvent^31^.

When the intra-polymer attraction is considered, such as *E*_PP_ = 1, the apparent exponent α becomes larger than that at *E*_PP_ = 0. At *E*_PP_ = 1, polymer becomes compact and the degree of compact increases with the chain length *N*. Therefore, the diffusion coefficient is further decreased with increasing *N*, resulting in a larger exponent α at larger *E*_PP_. For polymer on 2D surface, we find the simulation results can be well expressed by the scaling law *D*_xy_ ~ *N*^−α^ with α ≈ 1.66.

Whereas for the desorbed polymer in 3D dilute solution, the dependence of *D*_xy_ on *N* is of polymer length dependence at *E*_PP_ = 1 and 2. We find that *D*_xy_ decreases faster for short polymer length, i.e. the apparent exponent α estimated from short polymer region is larger. However, for long polymer region, it is interesting to see that the apparent exponent α is roughly the same as that of polymer on 2D surface. For partially adsorbed polymers, the diffusion coefficient lies between that of 3D and 2D cases. At *E*_PS_ = 1, we find the diffusion coefficient is close to the 3D one for short polymer lengths while that is close to the 2D one for long polymer lengths. While at *E*_PS_ = 2, the diffusion coefficient is close to 2D one even for short polymer lengths. Therefore we conclude that the apparent exponent α of the partially adsorbed long polymers is the same as that of polymer on 2D surface. A big value α ≈ 2.25 is estimated at *E*_PP_ = 2.

**Figure 3:**
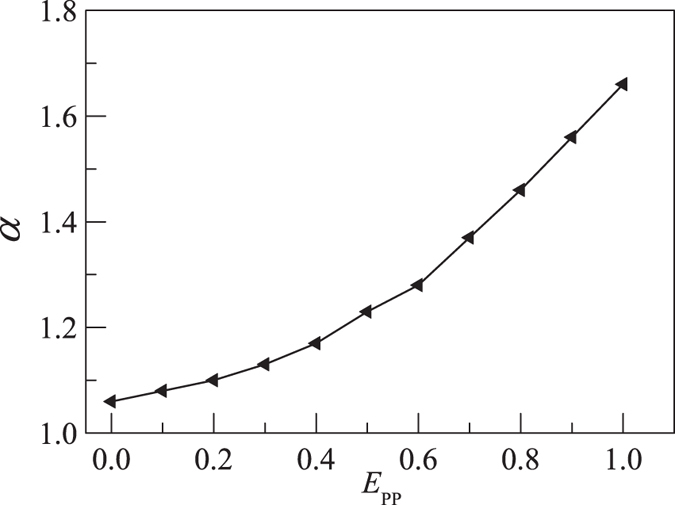
Plot of the apparent exponent α estimated from long polymers versus the intra-polymer attraction *E*_PP_. Simulation temperature *k*_B_*T* = 1.

Figure 2 shows that the apparent exponent α of long polymer is only dependent on *E*_PP_ but is independent of *E*_PS_. Figure 3 presents the apparent exponent α for the diffusion of long polymer. We find that α increases smoothly with *E*_PP_. As already shown in the above discussion, the decrease of *D*_xy_ with increasing *E*_PP_ is due to the fact that more and more monomers become close to each other. The longer the chain length is, the stronger the effect is. So the apparent exponent α increases with *E*_PP_.

In short, the diffusion of adsorbed polymer on the homogeneous surface is obviously slowed down by the attraction of surface. This is in agreement with experimental observations^22^,^25^,^51^ and other simulations^30^,^31^,^32^. Our results show that the exponent α in the scaling law *D*_xy_ ~ *N*^−α^ is independent of the surface attraction, however, this is inconsistent with experimental results^22^. We also observe a new result that α increases with the intra-polymer attraction.

We notice that the experiment on the surface diffusion of Poly(ethylene glycol) found that the scaling exponent α was increased obviously after adsorption^22^. The reason for such an increase is complex and is not clear^22^. Since our simulation results show that the homogeneous surface does not affect the scaling exponent α, one of the possible reasons for the change of α is that the surface in experiment is heterogeneous. A heterogeneous surface with non-uniformly distributed attraction points will produce a parallel barrier for the diffusion, and that may change the diffusion behavior as that of polymer in crowded environment. It was found that value α for the diffusion of DNA in agarose gels was increased obviously^51^. The diffusion of polymer on heterogeneous surface deserves further study. Another possible reason is that the hydrodynamic interaction (HI) effect, which was included in experiment, is not considered in the present work. A surface can change the HI effect because of less solvent molecules near surface. It will be also interesting to simulate the HI effect on the diffusion of adsorbed polymers in future.

### Rotational relaxation of partially adsorbed polymer

**Figure 4:**
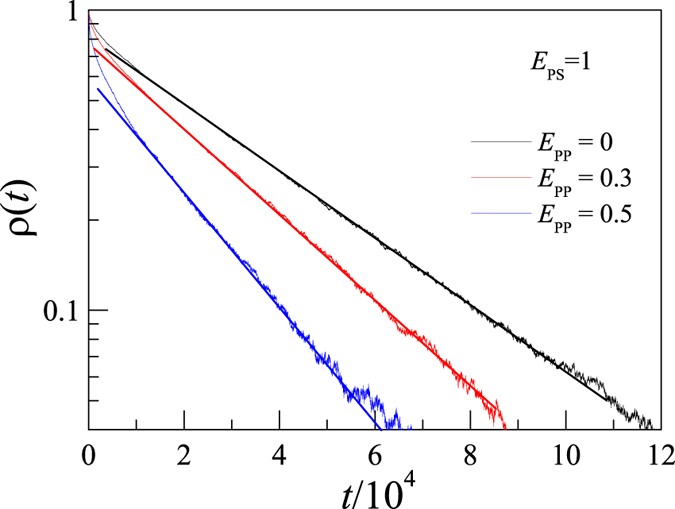
Semi-logarithm plot of end-to-end vector rotational autocorrelation function ρ(*t*) versus time for *N* = 64 at *E*_PS_ = 1 and different *E*_PP_. The solid lines are linear fits using the data in the region 0.06 < *ρ*(*t*) < 0.30.

In this subsection, we study the rotational relaxation of the partially adsorbed polymer chain on homogeneous surface. We have calculated the end-to-end vector rotational autocorrelation function of the polymer^29^,^30^,^52^,^60^


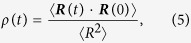


where ***R***(*t*) is the end-to-end vector of a polymer chain at time *t* and < > also means an average over the ensemble of independent samples. Here ρ(*t*) describes the degree of rotation for polymer. For the partially adsorbed polymer chain at temperature below CAP, the end-to-end distance vector ***R*** is roughly parallel to the surface, thus ρ(*t*) describes the rotation parallel to surface while the contribution from the surface normal direction can be neglected. It is expected that ρ(*t*) decays as


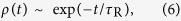


thus one can estimate the rotational relaxation time *τ*_R_ by fitting the decay of ρ(*t*) to an exponential decay function. Figure 4 presents the simulation results of ρ(*t*) for *N* = 64 at *E*_PS_ = 1 and *E*_PP_ = 0, 0.3, and 0.5. One can see that ρ(*t*) decays exponentially in a relatively long time region. However, the width of the exponentially decay region is dependent on the interaction parameters. In this work, *τ*_R_ is estimated from the decay of ρ(*t*) in the region 0.06 < ρ(*t*) < 0.30 where the best fitting can be always obtained^29^.

It is interesting to see that the rotational relaxation time *τ*_R_ of the partially adsorbed polymer decreases as the intra-polymer attraction *E*_PP_ increases. This is possibly because the intra-polymer attraction reduces the size of polymer. Then the moment of inertia of polymer for rotation, which is in proportion to the size of polymer, decreases with increasing *E*_PP_, too. Therefore the intra-polymer attraction accelerates the rotation of polymer. However, with further increase in *E*_PP_, the conformation of polymer chain will be changed from a random coil state to a compact globule state^53^. We find that it is difficult to estimate *τ*_R_ for polymer in the globule state at *E*_PP_ > 0.5. Therefore we restrict *E*_PP_ < 0.5 in this work.

**Figure 5:**
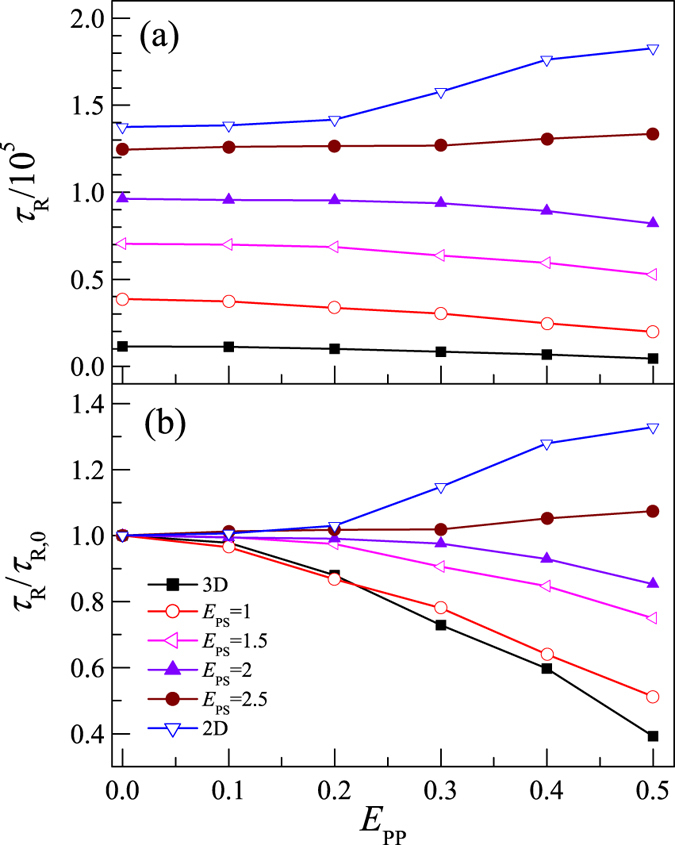
Plot of rotational relaxation time *τ*_R_ (**a**) and ratio *τ*_R_/*τ*_R,0_ (**b**) versus the intra-polymer attraction *E*_PP_ for polymer in 3D dilute solution, on 2D surface, and partially adsorbed on surface with polymer-surface attractions *E*_PS_ = 1, 1.5, 2, and 2.5. Polymer length *N* = 64. *τ*_R,0_ is the rotational relaxation time at *E*_PP_ = 0.

We have investigated the influence of surface attraction and intra-polymer attraction on *τ*_R_ for the partially adsorbed polymer. The values of *τ*_R_ at different *E*_PP_ and *E*_PS_ are simulated. Figure 5 presents the dependence of *τ*_R_ on *E*_PP_ for *N* = 64 at *E*_PS_ = 1, 1.5, 2, and 2.5. For comparison, results for polymer chains in dilute solution (desorbed polymer) and on 2D surface (fully adsorbed polymer) are also presented. We find that *τ*_R_ increases with *E*_PS_ for the partially adsorbed polymer, in agreement with previous simulation results^30^,^31^,^32^,^43^ and experiment^25^. The results show that the attractive surface obviously slows down the rotation of polymer.

It is interesting to notice that different behaviors of *τ*_R_ are observed for polymer in 3D dilute solution and on 2D surface, respectively. We find that *τ*_R_ decreases with an increase in *E*_PP_ in 3D dilute solution, but increases with *E*_PP_ on 2D surface. The result implies that the rotational behavior of polymer on surface is different from that in solution.

For the partially adsorbed polymer chain, *τ*_R_ is also dependent on *E*_PP_. Figure 5a shows that *τ*_R_ decreases with increasing *E*_PP_ at small *E*_PS_ but it increases with increasing *E*_PP_ at *E*_PS_ = 2.5. It is clear that the behavior of the partially adsorbed polymer changes from 3D like to 2D like with the increase in *E*_PS_. On the other hand, *τ*_R_ increases with *E*_PS_, the underlying reason could be explained as follows: the polymer-surface attraction keeps monomers close to the surface and makes it hard to relax rotationally. Figure 5b presents the ratio *τ*_R_/*τ*_R,0_ for different *E*_PS_, where *τ*_R,0_ is the rotational relaxation time at *E*_PP_ = 0. It is clear to see that the effect of surface on the polymer rotation increases with *E*_PS_.

We have calculated *τ*_R_ for other polymer lengths at different *E*_PP_ and *E*_PS_. For all polymer lengths, we observe that *τ*_R_ of the adsorbed polymer is larger than that in dilute solution, but smaller than the one on 2D surface. And for all *E*_PP_, *τ*_R_ increases monotonically with *E*_PS_. So the adsorbing surface not only reduces diffusion as discussed in the previous subsection, but also prevents the polymer from relaxing rotationally.

**Figure 6:**
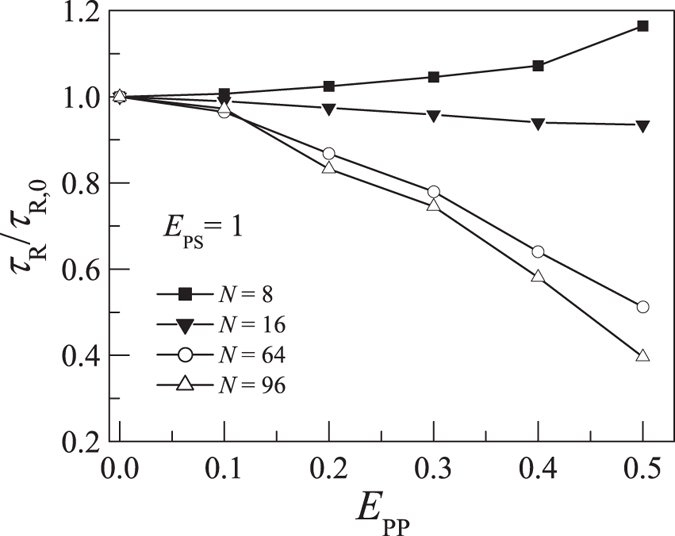
Plot of rotational relaxation time *τ*_R_ versus the intra-polymer attraction *E*_PP_ for polymers with length *N* = 8, 16, 64, and 96 with polymer-surface attraction *E*_PS_ = 1. *τ*_R,0_ is the rotational relaxation time at *E*_PP_ = 0.

From Fig. 5 we see that the behavior of the partially adsorbed polymer changes from 3D like to 2D like as the surface attraction strength *E*_PS_ increases. We have also simulated the rotational relaxation times for different polymer lengths from 8 to 128. For the partially adsorbed polymers, we find that the relationship between *τ*_R_ and *E*_PP_ is also dependent on the polymer length. Figure 6 presents the dependence of *τ*_R_ on *E*_PP_ for *N* = 8, 16, 64, and 96 at *E*_PS_ = 1. We find that *τ*_R_ decreases with increasing *E*_PP_ for long polymers but it increases with increasing *E*_PP_ for short polymers. It is known that, near CAP, the number of adsorbed monomers *M* increases with *N* as *M* ~ *N*^ϕ^ with the crossover exponent ϕ near 0.5^27^,^35^. Since the fraction of adsorbed monomers *M*/*N* ~ *N*^ϕ−1^ decreases with increasing polymer length, it is reasonable to find that long adsorbed polymers behave more like the polymer in dilute solution whereas short ones behave more like that on 2D surface. Shorter polymers are significantly influenced by the adsorbing surface and the corresponding *τ*_R_ increases with *E*_PP_.

**Figure 7:**
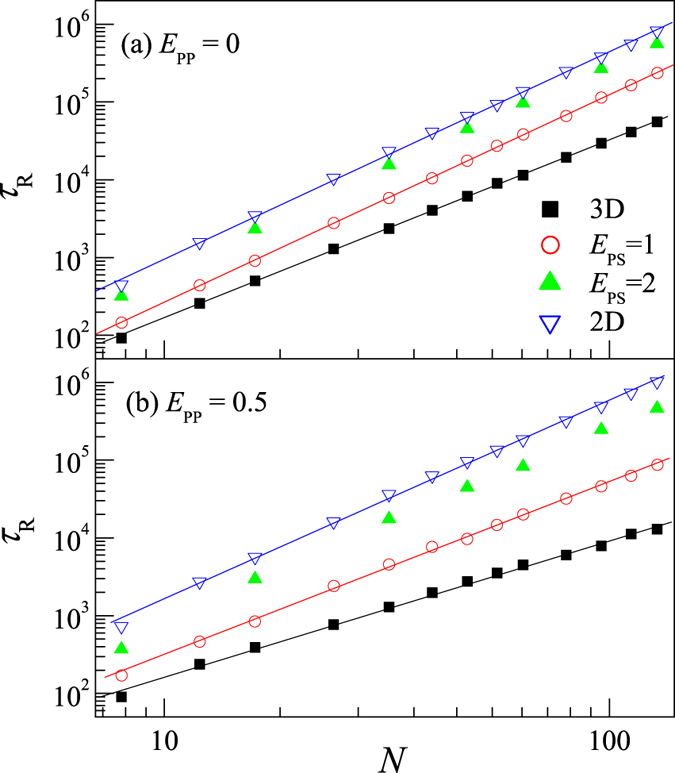
Log-log plot of *τ*_R_ versus *N* for partially adsorbed polymers on surface with *E*_PS_ = 1 and 2, desorbed polymer in 3D dilute solution, and fully-adsorbed polymer on 2D surface. Intra-polymer attraction is *E*_PP_ = 0 (**a**) and *E*_PP_ = 0.5 (**b**). Solid lines are guides for eyes.

Figure 7 presents the polymer length dependence of the rotational relaxation time *τ*_R_ for polymers in different situations. We find that the relaxation time can be always scaled as


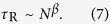


We estimate β = 2.3 for the simple SAW polymer (i.e. *E*_PP_ = 0) in dilute solution, indicating that the SAW polymer in dilute solution roughly follows Rouse dynamics with the exponent β = 1 + 2ν_3D_ = 2.2 with the Flory exponent ν_3D_ = 0.6. While we find β = 2.7 for the SAW polymer chain on 2D surface, also close to the expected value 1 + 2ν_2D_ = 2.5 with ν_2D_ = 0.75 for the dynamics of a SAW polymer on 2D surface. It is interesting to find that, for the partially adsorbed SAW polymer in the absence of intra-polymer attraction, i.e. *E*_PP_ = 0, the exponent β is roughly independent of the surface attraction and is roughly equal to that of a 2D SAW polymer, that is in agreement with earlier finding that β of a partially adsorbed polymer was independent of temperature^30^. Our value β = 2.7 for the adsorbed polymer is larger than 2.56 for a BF polymer model^32^ but close to β = 2.65 for an off-lattice polymer model^31^.

When the intra-polymer attraction is taken into account, for example at *E*_PP_ = 0.5, the scaling relation (Eq. 7) is still hold, but the exponent β is dependent on the surface attraction. We find β increases with *E*_PS_.

**Figure 8:**
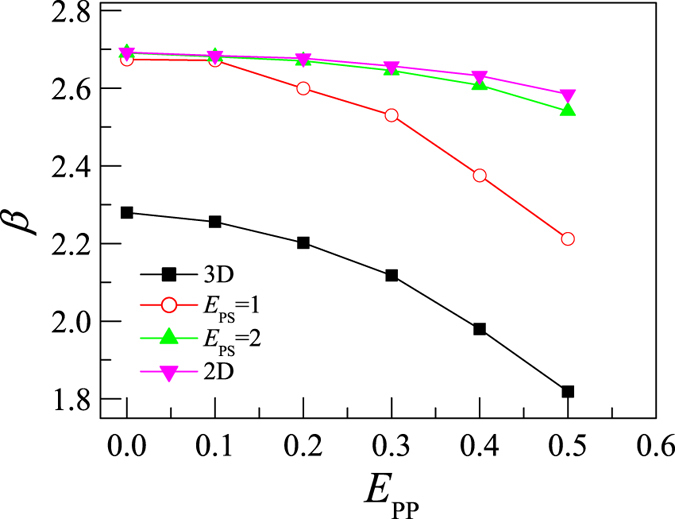
The exponent β plotted versus the intra-polymer attraction *E*_PP_ for partially adsorbed polymer with polymer-surface attractions *E*_PS_ = 1 and 2, desorbed polymer in 3D dilute solution, and fully-adsorbed polymer on 2D surface.

We find that the exponent β is dependent on both the surface attraction and the intra-polymer attraction. The exponent β at different *E*_PP_ and *E*_PS_ is calculated and the results are presented in Fig. 8. We find that β decreases with increasing *E*_PP_. The intra-polymer attraction attracts monomers together, that reduces polymer’s statistical size and accelerates the rotation. As the effect of the intra-polymer attraction on the polymer increases with polymer length, it is reasonable to find β decreases with increasing *E*_PP_. On the other hand, the surface will retard the rotation of polymer. Thus we find β increases with increasing *E*_PS_.

## Conclusions

**Figure 9:**
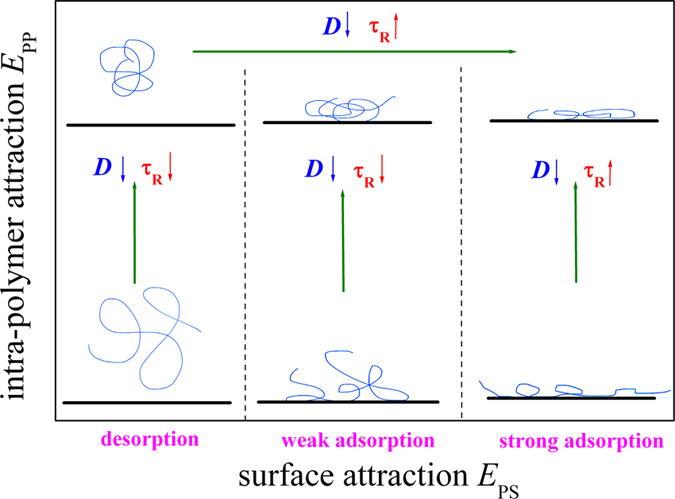
Sketch of polymer states and the dependence of diffusion coefficient *D* and rotational relaxation time τ_R_ on the intra-polymer attraction *E*_PP_ and the polymer-surface attractions *E*_PS_.

The dynamics of a bond fluctuation polymer chain adsorbed on an attractive, homogeneous surface is simulated by using dynamic Monte Carlo simulations. We have investigated the translational diffusion parallel to surface and the rotational relaxation of the partially adsorbed polymer. For comparison, we have also simulated the dynamics for a desorbed polymer chain and for a fully adsorbed one. Results show that the dynamics of polymer is strongly dependent on the intra-polymer attraction *E*_PP_ and the polymer-surface attraction *E*_PS_. However, the effects of the attractive surface on the diffusion and rotation of polymers are different. The main results are summarized in Fig. 9.

For the desorbed polymer in dilute solution, the translational diffusion is reduced whereas the rotation is speeded up with an increase in the intra-polymer attraction *E*_PP_.

The translational diffusion of polymer is reduced when it is adsorbed on surface. The translational diffusion coefficient *D*_xy_ of the adsorbed polymer also decreases with increasing *E*_PP_. The scaling exponent α of *D*_xy_ for long polymer length *N* is independent of *E*_PS_, i.e., it is the same for the desorbed polymer, partially adsorbed polymer, and fully adsorbed polymer. However α increases with *E*_PP_ from α = 1.06 at *E*_PP_ = 0.

The rotational relaxation time *τ*_R_ of the adsorbed polymer always increases with *E*_PS_, indicating that the surface attraction reduces the rotation of the adsorbed polymer. However, the dependence of *τ*_R_ on *E*_PP_ is dependent on the state of the adsorbed polymer. We find that, with an increase in *E*_PP_, *τ*_R_ decreases for the partially adsorbed polymer but increases for the fully adsorbed polymer. The scaling exponent β of *τ*_R_ with *N* is also dependent on *E*_PP_ and *E*_PS_. At *E*_PP_ = 0, we find β ≈ 2.7 independent of *E*_PS_ for the partially adsorbed polymer. But at *E*_PP_ > 0, we find β increases with *E*_PS_. However, β always decreases with increasing *E*_PP_.

## Additional Information

**How to cite this article**: Yang, Q.-H. and Luo, M.-B. Dynamics of adsorbed polymers on attractive homogeneous surfaces. *Sci. Rep.* **6**, 37156; doi: 10.1038/srep37156 (2016).

**Publisher’s note**: Springer Nature remains neutral with regard to jurisdictional claims in published maps and institutional affiliations.
